# Transcriptome analysis of salivary glands of rabies-virus-infected mice

**DOI:** 10.3389/fmicb.2024.1354936

**Published:** 2024-02-06

**Authors:** Xin Guo, Maolin Zhang, Ye Feng, Xiaomin Liu, Chongyang Wang, Yannan Zhang, Zichen Wang, Danwei Zhang, Yidi Guo

**Affiliations:** ^1^State Key Laboratory for Diagnosis and Treatment of Severe Zoonotic Infectious Diseases, Key Laboratory for Zoonosis Research of the Ministry of Education, Institute of Zoonosis, and College of Veterinary Medicine, Jilin University, Changchun, China; ^2^Changchun Veterinary Research Institute, Chinese Academy of Agricultural Sciences, Changchun, China; ^3^State Key Laboratory of Pathogen and Biosecurity, Beijing Institute of Microbiology and Epidemiology, Beijing, China

**Keywords:** rabies virus, salivary gland, parotid, transcriptome, mice

## Abstract

Rabies is a fatal zoonotic disease that poses a threat to public health. Rabies virus (RABV) is excreted in the saliva of infected animals, and is primarily transmitted by bite. The role of the salivary glands in virus propagation is significant, but has been less studied in the pathogenic mechanisms of RABV. To identify functionally important genes in the salivary glands, we used RNA sequencing (RNA-seq) to establish and analyze mRNA expression profiles in parotid tissue infected with two RABV strains, CVS-11 and PB4. The biological functions of differentially expressed genes (DEGs) were determined by Gene Ontology (GO) and Kyoto Encyclopedia of Genes and Genomes (KEGG) enrichment analysis, which revealed 3,764 DEGs (678 up-regulated and 3,086 down-regulated) in the CVS-11 infected group and 4,557 DEGs (874 up-regulated and 3,683 down-regulated) in the PB4 infected group. Various biological processes are involved, including the salivary secretion pathway and the phosphatidylinositol 3-kinase-Akt (PI3K-Akt) signaling pathway. This study provides the first mapping of the transcriptome changes in response to RABV infection in parotid tissue, offering new insights into the study of RABV-affected salivary gland function and RABV pathogenic mechanisms in parotid tissue. The salivary gland-enriched transcripts may be potential targets of interest for rabies disease control.

## 1 Introduction

Rabies is a zoonotic disease caused by the rabies virus (RABV), which belongs to the genus *Lyssavirus* of the family *Rhabdoviridae* (Fooks et al., 2017; Singh et al., 2017). Human rabies is almost invariably fatal once clinical symptoms occur. With a high global death count per year, rabies continues to pose a severe threat to human and animal health (Fooks et al., 2014). Initial symptoms of rabies include generalized symptoms such as fever, pain, and abnormal tingling or burning at the wound. As the virus spreads to the central nervous system (CNS), the disease progresses to fatal encephalitis and encephalomyelitis, leading to the development of the typical clinical signs of furious rabies or paralytic rabies and ultimately death from asphyxia caused by pharyngospasm or respiratory circulatory failure (Mitrabhakdi et al., 2005; Hemachudha et al., 2013; Jackson, 2016).

It has been reported that up to 99% of human rabies cases are caused by a deep bite or scratch from rabid dogs (Fooks et al., 2017). Cases of RABV infection from organ transplantation from infected patients and aerosol transmission are relatively rare (Srinivasan et al., 2005; Maier et al., 2010). Typically, following transmission by a deep bite from an infected animal, RABV replicates in muscle cells and binds to receptors at the neuromuscular junction, initiating early peripheral infection (Lewis et al., 2000). The virus travels through the peripheral nerve bypassing the spinal cord and reaches the CNS by centripetal spread. Once in the brain, RABV replicates on a large scale and spreads centrifugally to the peripheral nervous system, infecting surrounding tissues such as the heart, salivary glands, adrenal glands, gastrointestinal tract and pancreas (Hemachudha et al., 2002; Jogai et al., 2002; Dietzschold et al., 2008; Tobiume et al., 2009). The salivary gland is the primary outlet for the centrifugal spread of RABV and supports viral replication. Previous studies have shown that infected dogs can excrete viral particles in their saliva for up to 14 days before showing obvious clinical signs of rabies (Fekadu and Shaddock, 1984). The salivary gland epithelium contains a high concentration of RABV antigens and can be used as an alternative to brain and cerebrospinal fluid samples for RABV diagnosis (Beauregard and Casey, 1969; Dierks et al., 1969; Charlton et al., 1983).

Saliva is a unique oral bodily fluid. Approximately 90% of saliva is produced by the salivary glands, which mainly include the submandibular gland (SMG), sublingual gland (SLG), and parotid gland (PG) (Zhang et al., 2016a). The secretion of saliva is controlled by the cerebral cortex, and can be influenced by factors such as diet, environment, age, pathogen invasion and salivary gland lesions. Saliva contains various substances, including amylase, lysozyme, peroxidase, mucin, phospholipids, sodium, potassium, calcium, and magnesium, which contribute to its functions of food digestion, antibacterial activity, and protection of the gastric mucosa. Additionally, saliva has the ability to excrete certain pathogens such as RABV (Li et al., 1995) and enteric viruses like murine norovirus (MNV), rotavirus (RV) and astrovirus (AstV) (Kirby et al., 2010; Pisanic et al., 2019; Anfruns-Estrada et al., 2020; Ghosh et al., 2022). Epstein–Barr virus (EBV), herpes simplex virus (HSV), SARS-CoV-2 and human immunodeficiency virus (HIV) can also be excreted or transmitted through the saliva (Corstjens et al., 2000; Lee et al., 2009; Lima et al., 2010; Huang et al., 2021).

In this study, we utilized transcriptomics to characterize the mRNA expression in parotid tissues of mice infected with two RABV strains: the challenge virus standard (CVS)-11 strain and the street rabies virus PB4 strain, and we analyzed the biological functions of the differentially expressed mRNA. The accuracy of the RNA-seq data was confirmed by a quantitative real-time polymerase chain reaction (RT-qPCR). This research represents the first transcriptome analysis of salivary glands in RABV-infected mice and identifies potentially important salivary gland transcripts.

## 2 Materials and methods

### 2.1 Experimental animals

The Kunming mice used in this experiment were purchased from Liaoning Changsheng biotechnology co., Led. (License No. SCXK (Liao) 2015–0001). All mice were 3-week-old SPF Kunming mice, weighing approximately 15 g. Male and female mice were equally divided and provided with free access to food and water during the feeding period. A total of 36 mice were split into three groups: mock, CVS-11 and PB4-infected groups, with each group consisting of 12 mice. Within each group, four mice constituted a biological replicate.

### 2.2 Virus infection

The CVS-11 and PB4 strains used in this study were donated by Changchun Tu of the Diagnostic Laboratory of Rabies and Wildlife-associated Viral Zoonoses of Changchun Veterinary Research Institute, Chinese Academy of Agricultural Sciences. The PB4 strain was isolated from a rabid pig that was bitten by a rabid dog in Hunan province in 2006 by the National Reference Laboratory for Rabies in China. All virus-related experiments were conducted in a Biosafety Level III (BSL-3) laboratory, following the biosafety regulations of the International Guiding Principles for Biomedical Research Involving Animals. Mice were divided into two experimental groups, and each group was inoculated intramuscularly (into the right thigh muscle) with 100 μL of 10^5.5^ TCID50/mL of the CVS-11 strain or PB4 strain. A mock group was also included that was inoculated with an equal amount of Dulbecco’s Modified Eagle’s Medium (DMEM) (Sigma-Aldrich Co. LLC, St Louis, America). When the mice reached the late stage of the disease and died, their PGs were collected for detection and RNA sequencing.

### 2.3 RNA extraction and quantitative real-time PCR

Parotid tissues were lysed using TRIzol (Takara Biomedical Technology Co, Ltd., Kusatsu, Japan) for total RNA extraction. Subsequently, cDNA was synthesized using the All-In-One 5X RT MasterMix (Applied Biological Materials Inc., Richmond, Canada). RT-qPCR was conducted in a 20 μL reaction volume, consisting of 10 μL FastStart Universal SYBR Green Master Mix (ROX) (F. Hoffmann-La Roche Ltd., Basel, Switzerland), 1 μL PCR primers (containing 10 μM forward and reverse primers, respectively), 4 μL nuclease-free water, and 5 μL cDNA. The reference household gene glyceraldehyde-3-phosphate dehydrogenase (GAPDH) was utilized. Samples were incubated at 95°C for 10 min to activate DNA polymerase, followed by 40 cycles at 95°C for 15 s and at 60°C for 1 min for amplification and analysis. Primer sequences for the detection of RABV nucleoprotein (RABV N), GAPDH and other corresponding mRNAs are listed in Table 1. Each target was normalized to that of GAPDH using the 2^−ΔΔCt^ method.

**Table 1:** Primers used for validation of mRNA expression.

Gene name	Primer sequence
RABV-N Forward	TCAAGAATATGAGGCGGCTG
RABV-N Reverse	TGGACGGGCTTGATGATTGG
m-GAPDH Forward	TGTGTCCGTCGTGGATCTGA
m-GAPDH Reverse	TTGCTGTGAAGTCGCAGGAG
m-Acaca Forward	CCCAGAGATGTTTCGGCAGTCAC
m-Acaca Reverse	GTCAGGATGTCGGAAGGCAAAGG
m-Elovl6 Forward	TGCAGCATGACAACGACCAGTG
m-Elovl6 Reverse	AATGGCAGAAGAGCACAAGGTAGC
m-Lipo1 Forward	ATTAAGTTGGTTGATGCTGGATGCG
m-Lipo1 Reverse	CGGCTCGTATTTAATTGCTCTGGAG
m-Scgb2b7 Forward	TTGCTGGTGACTGGAGAACTGAG
m-Scgb2b7 Reverse	CAACCACACCCTACTTCCTGAGAG
m-Lcn11 Forward	CAATGGCAACCTGAATGTCACCTAC
m-Lcn11 Reverse	TGTGTCAGTCTTCTCTGCGATGTAG
m-Esp23 Forward	CTGCTGCTACTGTCAGTGCTCAC
m-Esp23 Reverse	ACGACCCTCTTGACATTTGTGCTG
m-Agr2 Forward	GGCGATCAGCTCATCTGGACTC
m-Agr2 Reverse	AGGCTTGACTGTGTGGGCATTC
m-Abo Forward	GACGGCAACTGGTGGTGCTAAC
m-Abo Reverse	GTCGCTCTGAGAAGTGGCTGATC
m-Cxcl13 Forward	TGTGTGAATCCTCGTGCCAAATGG
m-Cxcl13 Reverse	GAGCTTGGGGAGTTGAAGACAGAC
m-Phyh Forward	TTCCCCTTCCGACCTAGCAACC
m-Phyh Reverse	GCTTCAGAGTGCCTTTGTGGGTAC
m-Fasn Forward	CTCCTGAAGCCGAACACCTCTG
m-Fasn Reverse	AGCGACAATATCCACTCCCTGAATC
m-Esp31 Forward	GTTATCATCAATGTTCACCGAAGGAG
m-Esp31 Reverse	GAGGTTGAATGACATCTCAGGATTATG

### 2.4 RNA extraction library construction and sequencing

The quantity of total RNA was analyzed using the Bioanalyzer 2,100 and RNA 6000 Nano LabChip Kit (Agilent, CA, United States, 5067–1,511). High-quality samples with a RIN number > 7.0 were selected to construct the sequencing library. The mRNA was purified from the total RNA and cleaved into short fragments, which were then reverse-transcribed to generate the cDNA. Second-stranded DNAs were synthesized, and dual-index adapters were ligated to the fragments, whose size selection was performed using AMPureXP beads. The ligated products were amplified by PCR. Finally, 2 × 150 bp paired-end sequencing (PE150) was conducted on an Illumina Novaseq™ 6,000 (LC-Bio Technology CO., Ltd., Hangzhou, China).

### 2.5 Sequencing and filtering of clean reads

The cDNA library was sequenced and run with the Illumina Novaseq™ 6,000 sequencing platform. We employed the Illumina paired-end RNA-seq approach to sequence the transcriptome and generate 2 × 150 bp paired-end reads. The reads obtained from the sequencing machines include raw reads with adapters or low quality bases. A quality analysis was performed before sequencing and clean reads were filtered to check the quality of the sequencing data and rule out possible low-quality reads. We then filtered the reads with Cutadapt^1^ to obtain high quality clean reads. We utilized Q30, GC content, low-quality read content and other quality control indicators to evaluate data quality. During this process, we removed reads containing adapters, polyA and polyG, reads containing more than 5% of unknown nucleotides (N) and low quality reads containing more than 20% of low quality (Q-value ≤20) bases. We then verified the per- base sequence quality by using FastQC.^2^ As a result, a total of 47.92GB of cleaned, paired-end reads were produced. We successfully uploaded and published the clean raw sequence data to the NCBI Gene Expression Omnibus (GEO) datasets, under GEO accession number GSE248365.

### 2.6 Alignment to the reference genome

We aligned the reads of all samples to the reference genomes of *Mus musculus* (Ensembl release 101) using HISAT2.^3^ This first removes portions of the reads based on quality information and then maps the reads to the reference genome.

### 2.7 Quantification of gene abundance

The mapped reads of each sample were assembled with StringTie.^4^ We then merged all transcriptomes of all samples to construct a comprehensive transcriptome using gffcompare software.^5^ After the final transcriptome was generated, we estimated the expression levels of all transcripts and calculated the FPKM (fragment per kilobase of transcript per million mapped reads) value by using StringTie and ballgown^6^ for mRNA expression abundance analysis.

### 2.8 Differential gene expression analysis

Differential gene expression analysis was conducted between different groups using the DESeq2 software.^7^ Gene count was used as the input chosen for DESeq2. The fold change was calculated as the ratio of the mean expression value of the gene between the experimental groups and the mock groups. The genes with a false discovery rate (FDR) parameter below 0.05 and an absolute fold change (FC) ≥ 2 were considered as differentially expressed genes (DEGs).

### 2.9 Go and KEGG pathway enrichment analysis

Gene Ontology (GO) is a standardized classification system of gene functions. The basic unit of GO is a term corresponding to a specific attribute. KEGG is a prominent public database focused on pathways. Pathway enrichment analysis takes the KEGG pathways as a unit and applies a hypergeometric test to identify those that are significantly enriched in genes. DEGs with GO and KEGG annotations were enriched and analyzed. The number of genes was then calculated for each term, and GO and KEGG terms that showed significant enrichment in DEGs compared to the genomic background were identified using the hypergeometric test. The *p*-value is a statistical significance value used to measure the significance of DEGs. It is calculated within a hypothesis testing framework to assess the probability of observing the observed difference or a more extreme difference under the null hypothesis. The Q-value is a statistic used to control for the probability of false positive DEGs. It is a corrected *p*-value that can be used to assess whether DEGs are truly biologically significant. In GO and KEGG analyses, significantly enriched terms in DEGs were defined as those with a *p*-value less than 0.05 for GO and KEGG terms.

## 3 Results

### 3.1 RNA identification and classification

**Figure 1:**
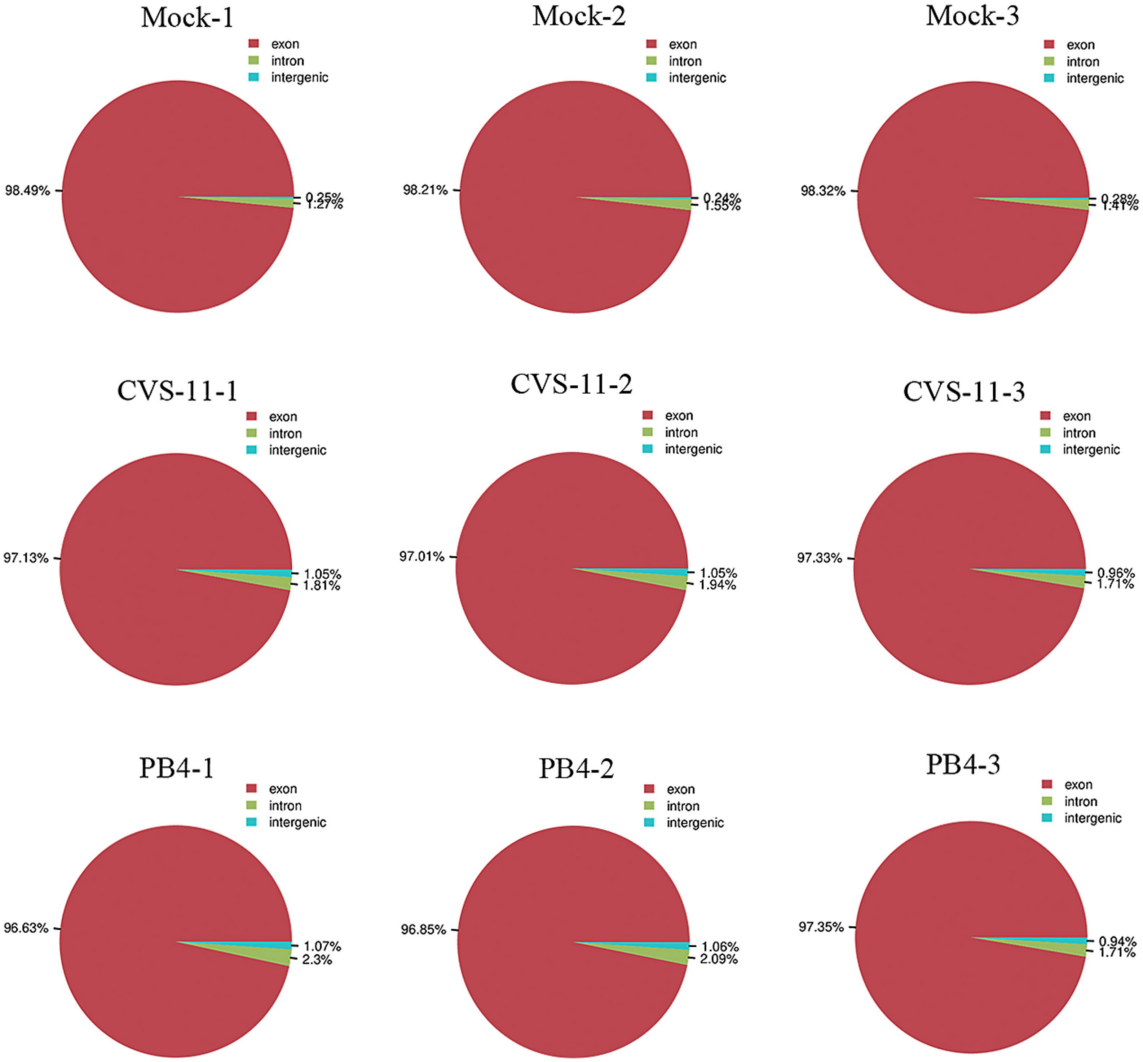
Pie chart showing the distribution of RNA on the chromosomes in parotid glands of RABV-infected and mock groups. Valid data are derived from the exon, intron and intergenic region.

To obtain the mRNA expression change profiles caused by RABV infection, a transcriptomic approach was performed using salivary gland tissue from mice infected with CVS-11 and PB4 strains, as well as a mock group. The RABV-infected mice exhibited clinical signs of infection, such as hunchback, collapse, tremors and hind limb paralysis as early as 3–4 days post-infection (dpi). At the advanced stage of the disease, we collected tissues from the SMG, SLG, and PG to measure the viral load using RT-qPCR. Our results showed that PG had the highest viral load compared to SMG and SLG (SI Figure 1). Therefore, our transcriptomic study focused on the parotid gland. We identified a total of 353,926,970 raw data points in all three groups, and the sequencing quality met the required standards (Table 2). The distribution of RNA on the chromosomes of the RABV- and mock-infected groups is shown in the pie chart (Figure 1). Valid data were derived from exons, introns, and intergenic regions.

**Table 2:** Sample quality control statistics.

Sample	Raw data	Valid data	Valid ratio	Q20%	Q30%	GC content%
Mock-1	38,758,916	34,266,866	88.41	99.98	98.41	47.00
Mock-2	37,505,828	33,619,778	89.64	99.97	97.97	46.50
Mock-3	36,783,676	33,266,800	90.44	99.98	97.97	46.50
CVS-11-1	43,766,134	38,844,936	88.76	99.98	98.35	43.00
CVS-11-2	40,510,116	39,901,008	98.50	99.98	98.21	42.00
CVS-11–3	38,579,116	34,230,276	88.73	99.99	98.47	42.00
PB4-1	37,716,318	32,618,430	86.48	99.99	98.61	42.50
PB4-2	39,292,932	38,396,372	97.72	99.99	98.50	41.50
PB4-3	41,013,934	34,306,576	83.65	99.99	98.81	42.00

### 3.2 Transcriptome regulation of parotid tissue after RABV infection

**Figure 2:**
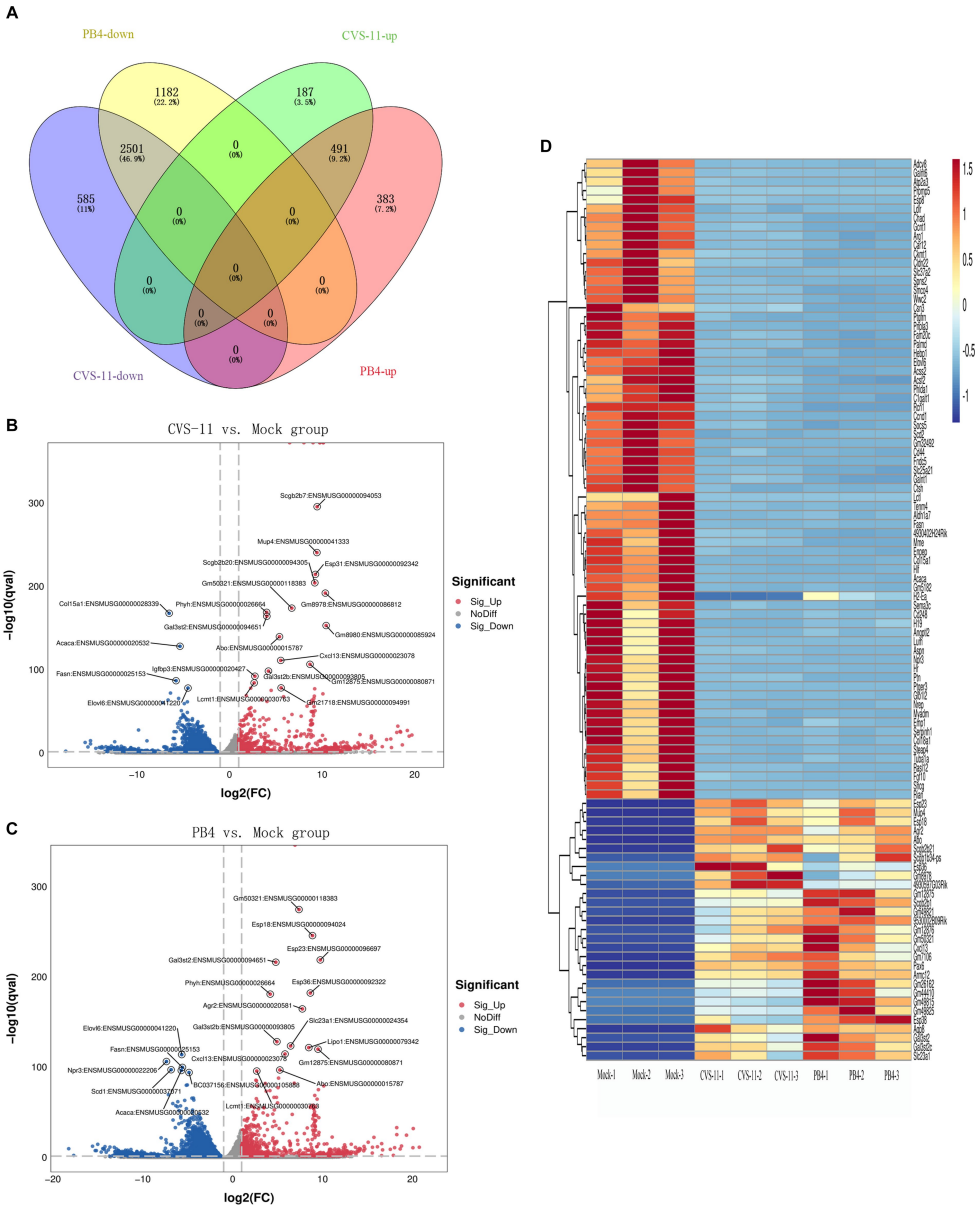
Overall distribution of DEGs. **(A)** Venn diagram showing the intersection of DEGs detected in the RABV-infected and mock groups. **(B,C)** The volcano plot shows the distribution of DEGs, with log2 (FC) as the abscissa and -log10 (Q-value) as the ordinate (showing the Top 20 genes with the lowest Q-value). **(D)** Heatmap showing the clustering analysis of genes according to the similarity of gene expression profiles of samples in the RABV-infected and mock groups, with samples as the abscissa and DEGs as the ordinate (showing the Top 100 genes with the lowest Q-value). The scale represents the data distribution range of the absolute value of the FPKM of genes after Z-Score standardization. All the DEGs were consistent with |log2FC| ≥ 1&q < 0.05.

We identified a total of 3,764 DEGs (678 up-regulated and 3,086 down-regulated) in the CVS-11- infected group. Similarly, we found 4,557 DEGs in the PB4- infected group, with 874 genes up-regulated and 3,683 genes down-regulated. The top 20 up- and down-regulated DEGs with the largest fold change in the CVS-11 and PB4-infected parotid glands compared to the mock group are shown in Tables 3, 4. The intersection of DEGs between the mock group and the experimental group was visually shown using the Venn diagram (Figure 2A). Furthermore, we utilized the volcano map (Figures 2B,C) and the heat map (Figure 2D) to depict the distribution of DEGs. Our findings demonstrate that both CVS-11 and PB4 infection significantly alter the mRNA expression profile in the mouse parotid gland, leading to the dysregulation of multiple genes.

**Table 3:** Top 20 up- and down-regulated DEGs in the CVS-11 infected parotid gland.

Up		Down	
Gene name	Fold change	Gene name	Fold change
Scgb1b18	2517509.91666667	Amy2b	4.76158014306803E-06
Gm12876	855961.976666667	Gm32492	0.0000203404323208901
Gm21903	697076.916666667	Mup16	0.0000250786628541530
Scgb1b34-ps	607236.936666667	Lctl	0.0000304048922861626
Scgb1b11	417606.760000000	Gm7030	0.0000304118343542707
Scgb1b19	367812.190000000	Wfdc21	0.0000452004981094892
Scgb2b21	354537.370000000	Gm49450	0.0000584366672166206
Aqp8	298321.678625343	Crabp1	0.0000669635831945513
Scgb2b23-ps	275966.668118467	Prss34	0.0000710450634100873
Scgb1b28-ps	185361.026666667	Klk1b27	0.0000770479934519479
Gm49815	175944.906666667	Ighv14-2	0.0000912133288213140
Gm29748	128881.453333333	Gm31378	0.0000948348814074002
Scgb1b4-ps	93043.4300000000	C1qtnf5	0.0000959793721930592
Gm19724	55032.5433333333	Gm50341	0.0001084663793994870
Scgb2b29-ps	53802.2433333333	Gm5144	0.0001086305069877630
Scgb2b18	53534.5200000000	Smr2	0.0001148924198792910
Gm47809	46591.7233333333	Gm15564	0.0001154011161331700
Gm49822	43394.0500000000	Rarres1	0.0001156355890881630
Armc12	41361.0247332078	Gm28156	0.0001175437586027340
Gm13539	40932.3375686275	Klk1b26	0.0001276995688862550

**Table 4:** Top 20 up- and down-regulated DEGs in the PB4 infected parotid gland.

Up		Down	
Gene name	Fold change	Gene name	Fold change
Scgb1b18	1771929.04333333	Rps18-ps4	3.23946144342239E-06
Gm12876	1113274.58666667	Amy2b	4.76158014306803E-06
Scgb1b11	1084193.82666667	Gm32492	0.0000203404323208901
Gm49815	661505.683333333	Mup16	0.0000250786628541530
Scgb1b34-ps	417606.760000000	Gm7030	0.0000304118343542707
Scgb1b19	451161.470000000	Frmpd1os	0.0000272537363736996
Scgb1b28-ps	407166.120000000	Lctl	0.0000304048922861626
Aqp8	311876.605005271	Areg	0.0000448163604812919
Ly6g2	264903.508320739	Gm6522	0.0000477709136282773
Gm28116	240823.040000000	Upk3a	0.0000506112233086952
Scgb1b25-ps	190305.430000000	Kera	0.0000531218658099172
Scgb1b19	104748.810000000	Gm49450	0.0000584366672166206
Gm29748	97595.5333333333	C330024C12Rik	0.0000686138672285075
Scgb2b23-ps	96993.9238675957	Gm27544	0.0000751168066343164
Gm19724	70495.3233333333	Rtn4rl1	0.0000781003699093854
Armc12	53875.6553609542	Gm31378	0.0000948348814074002
Scgb2b22-ps	48058.7900000000	Gm50341	0.0001084663793994870
Gm21903	47430.4166666667	Fgf13	0.0001089791194347670
Gm41506	34601.8600000000	Gm12121	0.0001106536198671940
Gm47809	26641.2700000000	Gm45062	0.0001107696124983570

### 3.3 Functional enrichment analysis of mice PG transcriptome

**Figure 3:**
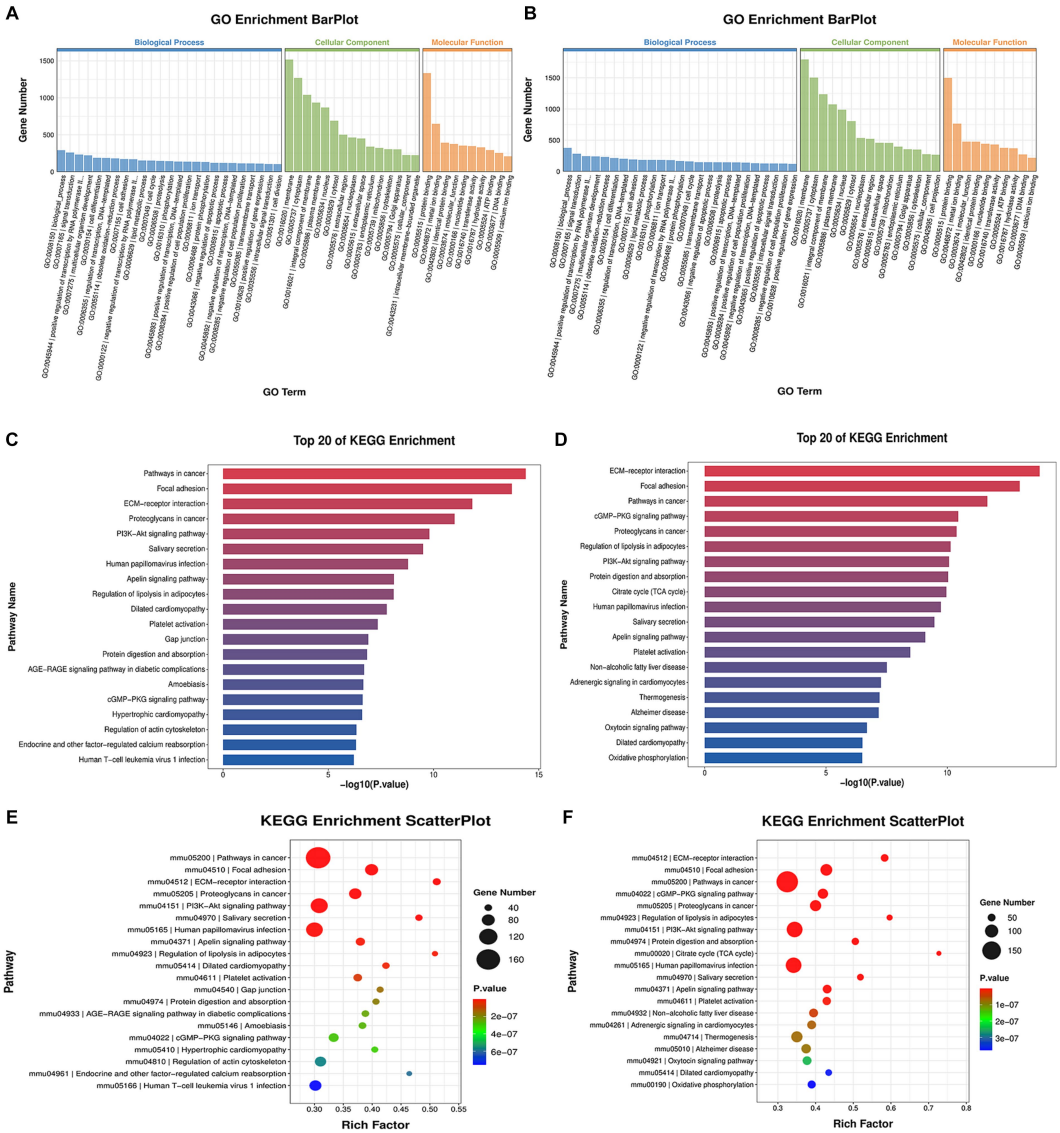
GO and KEGG enrichment analysis. **(A**,**C,E)** showing the analysis results of the CVS-11-infected group, with **(B,D,F)** showing those of the PB4-infected group. **(A,B)** The histogram of the GO enrichment classification reflects the distribution of DEGs in terms of enrichment. Top 25, Top 15 and Top 10 terms in BP, CC and MF are sorted from largest to smallest, respectively, according to the number of DEGs noted, with GO terms as the abscissa and the number of DEGs as the ordinate. **(C,D)** The histogram of the KEGG enrichment classification showing the first 20 pathways with the lowest *p*-value. The ordinate is the pathway name, and the abscise is the -log10 (*p*-value) of the corresponding KEGG terms. **(E,F)** KEGG enrichment scatterplot displaying the KEGG enrichment analysis results in the form of a bubble diagram by taking the Top 20 pathways with the smallest value of *p*, where the abscissa represents the proportion of the number of DEGs in the total number of genes located in that pathway, and the ordinate represents the KEGG pathway. The size of the bubble represents the number of genes, and the color of the bubble represents the p-value of the enrichment analysis, that is, the significance of the enrichment. The *p*-value in **E** ranges from 2e-07 to 6e-07, and that in **F** ranges from 1e-07 to 3e-07.

To gain insight into the potential function of the aforementioned DEGs, we conducted GO and KEGG analyses. The GO enrichment analysis revealed that both infected groups showed similar enrichment patterns (Figures 3A,B). The ‘biological processes’ were mainly enriched in signal transduction, positive regulation of transcription by RNA polymerase II and cell differentiation. The ‘cell component’ was mainly enriched in the cell membrane, cytoplasm and integral component of the membrane. The ‘Molecular functions’ included protein binding, metal ion binding and identical protein binding. KEGG enrichment analysis showed similar pathways in both infected groups, with enrichment in pathways such as cancer, focal adhesion, the PI3K-Akt pathway, the salivary secretion, the cyclic guanosine monophosphate-protein kinase G (cGMP-PKG) signaling pathway, and the extracellular matrix (ECM)-receptor pathway (Figures 3C–F). We focused on pathways related to classical RABV infection signaling pathways or salivary gland functions for further analysis, which are described in detail below.

### 3.4 Associated crucial signaling pathways

**Figure 4:**
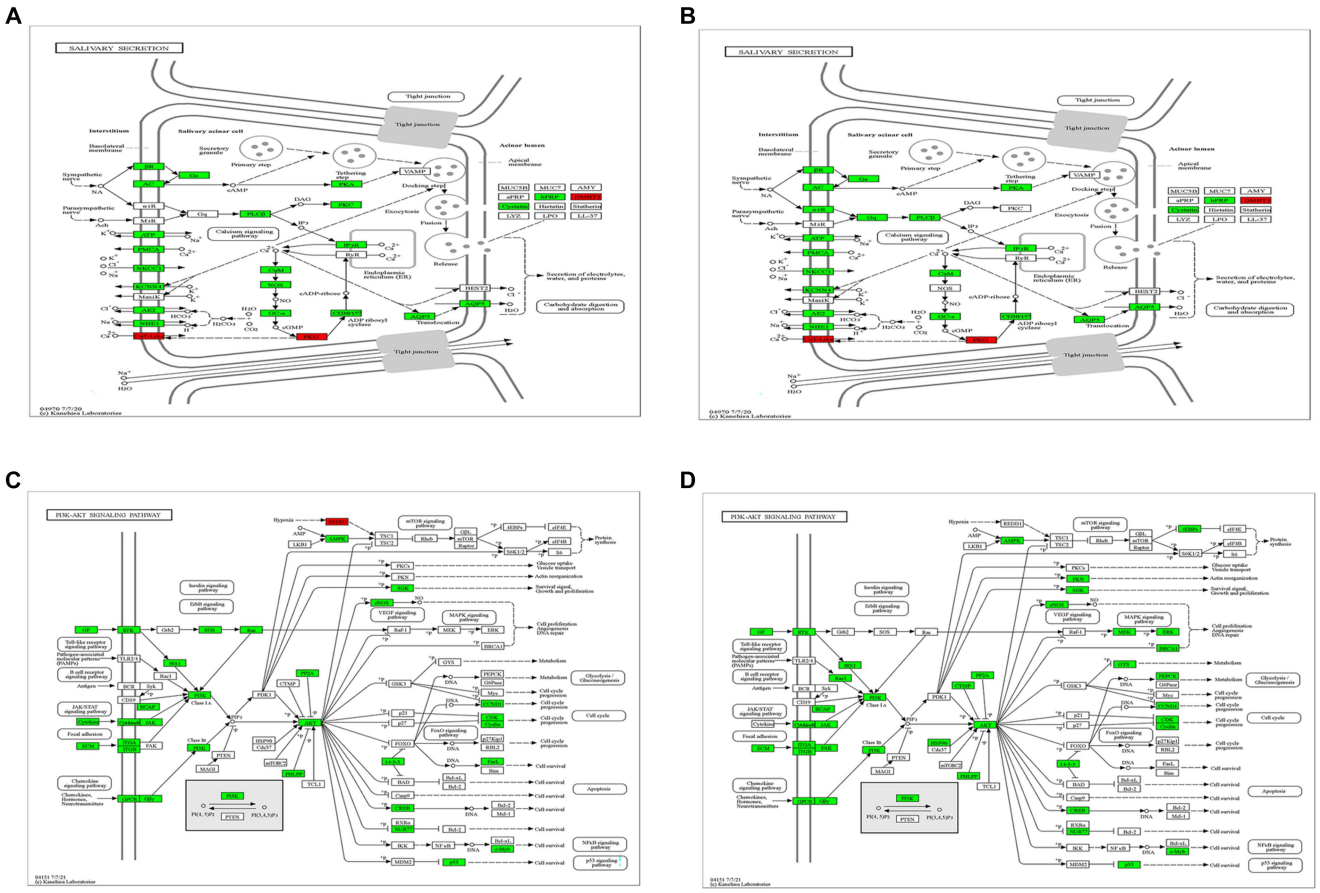
Crucial KEGG pathway maps. Changes in gene expression of the salivary secretion pathway in the CVS-11-infected group **(A)** and the PB4-infected group **(B)**, and that of the PI3K-Akt pathway in the CVS-11-infected group **(C)** and the PB4-infected group **(D)** are shown. The up-regulated genes are marked in red and the down-regulated genes are in green. The genes highlighted in the discussion are marked in the red frame.

CVS-11 or PB4 infection leads to thousands of DEGs in mouse PG tissue, and the DEGs are highly enriched in the salivary secretion pathway and the PI3K-AKT pathway. RABV was found to replicate in the salivary gland, and the physiology of this tissue in turn affects viral secretion. Figures 4A,B display some of the DEGs involved in the salivary secretion pathway. Our sequencing data revealed that 6 genes were up-regulated and 32 genes were down-regulated in the CVS-11-infected group, while 5 genes were up-regulated and 36 genes were down-regulated in the PB4-infected group. Among them, *aquaporin 5 (AQP5)*, *K^+^ channel protein-potassium calcium-activated channel subfamily N member 4 (KCNN4)*, and *ADP-ribosyl cyclase (ADPRC)/cyclic ADP-ribose (cADPR) hydrolase 1 (CD38)* were significantly down-regulated in both CVS-11 and PB4-infected samples, whereas *the agglutinin deleted in malignant brain tumor 1 (DMBT1)* showed obvious up-regulation in both RABV-infected groups. The majority of proteins encoded by DEGs in this pathway are Na^+^/K^+^-ATPase (NKA) subunits, ATPase, adrenergic receptor, adenylate cyclase, and inositol 1,4,5-trisphosphate receptor. Besides, DEGs in the parotid gland were significantly enriched in the PI3K-AKT pathway (Figures 4C,D), which is a frequently targeted intracellular signal transduction pathway by various viruses, including the mouse polyomavirus (MPyV), simian virus 40 (SV40) and papillomaviruses (PVs) (Buchkovich et al., 2008; Saeed et al., 2008; Feng et al., 2011). In the CVS-11-infected group, 111 genes were significantly enriched in this pathway (2 up-regulated and 109 down-regulated genes), while the PB4-infected group included 124 genes that were significantly enriched in this pathway (2 up-regulated and 122 down-regulated genes).

### 3.5 Validation of differentially expressed mRNAs by RT-qPCR

**Figure 5:**
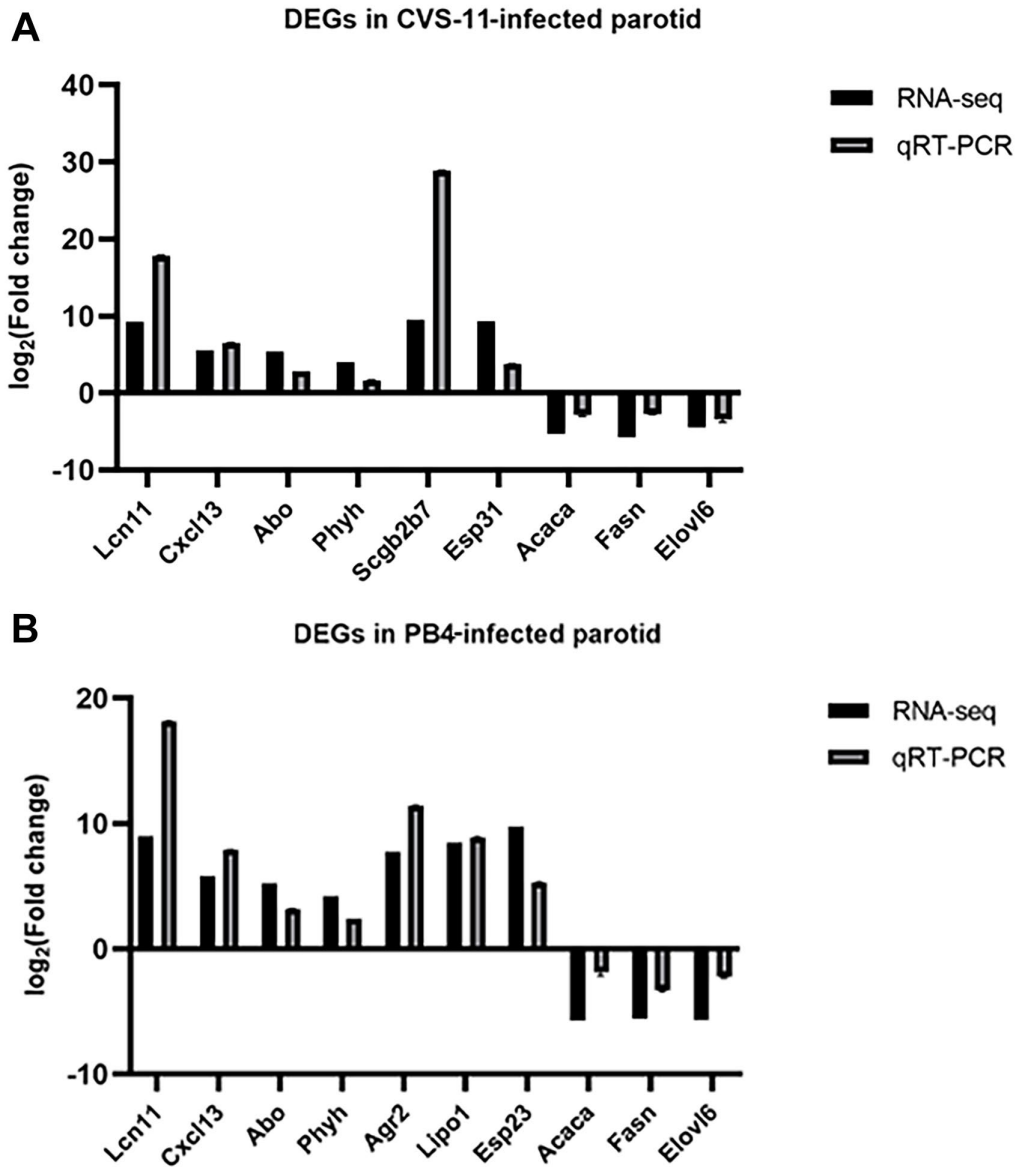
RT-qPCR validation of DEGs. Different primers were used for RT-qPCR to detect the expression changes of significantly regulated mRNAs in the CVS-1- infected group **(A)** and the PB4- infected group **(B)** compared to the mock group in the RNA sequencing data. The abscissa indicates the gene name, and the ordinate represents the value of log2 (FC). The expression of all genes was normalized to the housekeeping gene of GAPDH.

To validate the reliability of the RNA-seq data, we performed RT-qPCR to detect DEGs in RABV-infected and mock samples. We selected some of the Top 20 genes with the lowest Q-value that exhibited significant changes in CVS-11 and PB4-infected samples compared to the mock group for RT-qPCR validation (Figures 5A,B). The RT-qPCR results showed high concordance between the two datasets. The expression of *lipocalin 11 (Lcn11)*, *C-X-C motif chemokine ligand 13 (Cxcl13)*, *alpha 1-3-N-acetylgalactosaminyltransferase and alpha 1-3-galactosyltransferase (Abo),* and *phytanoyl-CoA 2-hydroxylase (Phyh)* were upregulated, while *acetyl-CoA carboxylase alpha (Acaca), fatty acid synthase (Fasn),* and *ELOVL fatty acid elongase 6 (Elovl6)* were significantly decreased in both strain-infected groups. Additionally, several genes were selected that showed significant trends of change in each group. *Secretoglobin, family 2B, member 7 (Scgb2b7)* and *exocrine gland secreted peptide 31 (Esp31)* were found to be increased in CVS-11 infected-parotids, while *exocrine gland secreted peptide 23 (Esp23)*, *anterior gradient 2 (Agr2)* and *lipase member O1 (Lipo1)* were down-regulated in PB4-infected parotids. Although the multiples of change are not necessarily very similar, the RT-qPCR results exhibited similar variation trends with the transcriptomic data in the infected groups compared to the mock group, suggesting that the results of the RNA-seq datasets are reliable.

## 4 Discussion

Rabies is a highly fatal zoonotic disease that is primarily transmitted through the bites of infected animals, like dogs in many developing regions of Asia and Africa, and wild animals such as bats and foxes in Western Europe and the Americas (Wilde et al., 2013; Anderson and Shwiff, 2015). Rabies often progresses to a fatal form of encephalomyelitis (Hemachudha et al., 2013). It exhibits neurophilic properties and causes damage to the nervous system (Hemachudha et al., 2013). Salivary excretion of the virus is necessarily influenced by the basic physiology of the salivary gland (Boonsriroj et al., 2016). Transcriptomics has been widely used to screen for potentially functional genes. Previous RABV-related transcriptomics studies have mainly focused on the nervous system, investigating changes in lncRNA, circRNA, and mRNA expression in the mouse brain caused by RABV infection (Zhang et al., 2016b; Sui et al., 2021; Zhao et al., 2021). The spatial and cellular distribution of RABV infection in the mouse brain has also been deeply revealed (Huang and Sabatini, 2020; Zhang et al., 2022). However, studies on the pathogenic mechanism of RABV rarely focus on the salivary glands. To further examine the mechanism of salivary gland function, we infected mice with CVS-11 and PB4 strains and performed RNA sequencing of the PG for the first time. We identified 3,764 DEGs (678 up-regulated and 3,086 down-regulated) in the CVS-11 infected group, and 4,557 DEGs (874 up-regulated and 3,683 down-regulated) in the PB4 infected group.

The salivary glands are mainly composed of SMG, SLG, and PG, along with minor salivary glands (MSG) scattered throughout the mouth. Among these glands, 85–90% of the saliva is produced by the PG and SMG. The PG is located on each side of the face anterior to the ears, and is the main source of stimulated salivary secretion, secreting watery saliva that plays a role in lubricating the oral cavity and maintaining homeostasis. Actions such as biting and chewing are accompanied by contraction of the masseter muscle, which leads to secretion of saliva from the PG into the oral cavity (Brazen and Dyer, 2023). RABV has been found to invade and replicate in the PG of infected hosts (Howard, 1981; Charlton et al., 1984; Silva et al., 2009), and it is possible that a bite from a rabid animal may stimulate the secretory function of the PG. The process of fluid secretion in the salivary glands relies on various ion-transporting proteins expressed in the acinar cells. These proteins are primarily targeted at the apical or basolateral membrane of the acinar cells. In the salivary secretion pathway, several genes that regulate the transport of water and important ion-transporting proteins across membranes were found to be dysregulated in our RABV-infected samples. *AQP5*, a protein primarily expressed in exocrine glands like salivary and lacrimal glands, plays a crucial role in water transport and is decreased following RABV infection (Agre et al., 1993; Agre, 2004). *AQP5* is critical for maintaining the normal physiology of the salivary glands, and its expression in salivary gland tissue could be affected by sialolithiasis and chronic salivary gland inflammation (D'Agostino et al., 2020). Research in *AQP5* mutant rats (Murdiastuti et al., 2006), *AQP5* knockout mice (Ma et al., 1999), and Sjögren’s syndrome patients (Steinfeld et al., 2001; Ichiyama et al., 2018; Hosoi et al., 2020) has demonstrated that abnormal expression of *AQP5* leads to decreased salivary secretion and hypertonic and viscous saliva, which induces thirst or dryness in the oral cavity. Additionally, AQP5 has been found to be associated with viral infections. For instance, PRRSV and adenovirus infection could lead to the downregulation of *AQP5*, hindering water clearance and edema resolution, and causing abnormal fluid flux during pulmonary inflammation in infected animals (Generous et al., 2014; Zhang et al., 2018). The reduction of *AQP5* expression in the infected parotid glands suggests that RABV may inhibit the expression and function of *AQP5*, thus affecting the intracellular and extracellular fluid balance and the function of water channels. This may be related to the invasion and spread of RABV and the development of the disease. *CD38* is a transmembrane glycoprotein that acts as a Ca^2+^ releaser from intracellular Ca^2+^-stores (Lee, 1997; Lee, 2001). Furthermore, the Ca^2+^-activated K^+^ channel of the intermediate single channel conductance encoded by *KCNN4* has been identified in exocrine salivary glands (Catalán et al., 2014). In our study, *CD38* and *KCNN4* were both decreased in mRNA levels, suggesting that RABV may affect the ion transporting system in salivary glands.

There are also dysregulated sequenced genes involved in the salivary secretion signaling pathway. *DMBT1*, which belongs to the scavenger receptor cysteine-rich (SRCR) superfamily (Generous et al., 2014; Reichhardt et al., 2017) and interacts with various pathogens in saliva (Ligtenberg et al., 2007), was significantly upregulated in both CVS-11 and PB4-infected groups. This may be due to the increased agglutination of virions in the salivary glands. *NOS* generates nitric oxide (NO) which is thought to contribute to protective and cytotoxic antiviral responses (Wink et al., 1996). *Inducible nitric oxide synthase (iNOS)* expression and activity have been shown to increase markedly in the brain of rats infected with RABV, indicating its importance in rabies neuropathogenesis (Hooper et al., 1995; Van Dam et al., 1995). *Endothelial nitric oxide synthase (eNOS)* expression was increased in cerebellar Purkinje cells and brainstem neurons in the brains of rabid cattle. Furthermore, *eNOS* and RABV were observed to co-localize in Negri bodies, suggesting the involvement of *eNOS* in the formation of RABV inclusion bodies (Akaike et al., 1995). *NOS* is widely distributed in various parts of the salivary glands, and is probably activated leading to the generation of cGMP, which opens ion channels to initiate the salivary secretory process (Bi and Reiss, 1995; Lomniczi et al., 1998). In our sequencing results, we observed down-regulation of *NOS* in the RABV-infected group, indicating that RABV may cause salivary gland damage similar to autoimmune inflammation, resulting in decreased *NOS* and NO expression levels to facilitate viral infection. Moreover, other signaling pathways are also involved in CVS-11 and PB4 infection, like the PI3K-Akt signaling pathway, the *janus kinase (JAK)*-*signal transducer and activator of transcription (STAT)* signaling pathway, and the *retinoic acid-inducible gene I (RIG-I)*-like receptor signaling pathway. Although it has been reported that RABV could stimulate these immune signaling pathways (Der et al., 1998; Goodbourn et al., 2000; Hornung et al., 2006; Faul et al., 2010), most immunity-related genes in our transcriptome data was restrained, like *interferon gamma receptors (Ifngrs)*, *interferon alpha receptors (Ifnars)*, *Janus kinases (Jaks)*, *signal transducers and activators of transcription (Stats)*, *stimulator of interferon response cGAMP interactors (Stings)*, and *TNF receptor associated factor 5 (Traf5)*. It has been speculated that the host immune system remains in a suppressed state as rabies progresses to late-stage disease, in order to persist the transmission and secretion of virions through saliva. However, the detailed mechanism by which the genes regulating salivary virion production and salivary gland function are dysregulated remains unclear. Further research is needed. Whether these genes could be potential targets for blocking virus transmission through saliva requires further validation. Whether the gene expression map of parotid tissue in the early stage of rabies differs from that in the later stage may merit intensive identification.

In summary, we demonstrated differentially expressed mRNA affected by RABV infection in the parotid tissues by employing the transcriptomic technique, and established GO and KEGG functional enrichment analyses. It is revealed that RABV invasion caused drastic regulation of the saliva secretion process in mice PG along with various host response processes. This study was the first to analyze the gene expression profiles and biological functions of the DEGs derived from RABV-infected mouse salivary tissues, which may have implications for the development of therapeutics, diagnostics and sanitation measures aimed at preventing the spread of rabies through saliva.

## 5 Conclusion

RNA-seq was utilized to establish mRNA expression profiles in PG mice infected with CVS-11 and PB4, and the biological functions of DEGs were analyzed. GO and KEGG enrichment analyses showed that most of the dysregulated genes were associated with the salivary secretion pathway, the PI3K-Akt signaling pathway, and the cGMP-PKG signaling pathway. Although some work has been undertaken, the detailed mechanism underlying RABV-induced salivary gland dysfunction remains elusive. These findings will contribute to a deeper understanding of the biology of viral spread.

## Data availability statement

The datasets presented in this study can be found in online repositories. The names of the repository/repositories and accession number(s) can be found in the article/Supplementary material.

## Ethics statement

The animal study was approved by the Institutional Animal Care and Use Committee of Jilin University. The study was conducted in accordance with the local legislation and institutional requirements.

## Author contributions

XG: Formal analysis, Investigation, Methodology, Validation, Writing – original draft. MZ: Conceptualization, Funding acquisition, Project administration, Writing – review & editing. YF: Funding acquisition, Investigation, Validation, Writing – review & editing. XL: Formal analysis, Investigation, Writing – review & editing. CW: Formal analysis, Investigation, Writing – review & editing. YZ: Formal analysis, Investigation, Writing – review & editing. ZW: Formal analysis, Investigation, Writing – review & editing. DZ: Investigation, Writing – review & editing. YG: Conceptualization, Funding acquisition, Investigation, Project administration, Supervision, Writing – original draft, Writing – review & editing.

## Footnotes

^1^https://cutadapt.readthedocs.io/en/stable/, version: cutadapt-1.9.

^2^http://www.bioinformatics.babraham.ac.uk/projects/fastqc/, version: 0.11.9.

^3^https://daehwankimlab.github.io/hisat2/, version: hisat2-2.2.1.

^4^http://ccb.jhu.edu/software/stringtie/, version: stringtie-2.1.6.

^5^http://ccb.jhu.edu/software/stringtie/gffcompare.shtml, version: gffcompare-0.9.8.

^6^http://www.bioconductor.org/packages/release/bioc/html/ballgown.html, version: 2.12.0.

^7^https://bioconductor.org/packages/release/bioc/html/DESeq2.html, version: 1.22.2/3.22.5.
