# Supplementary material for: Transcriptome analysis of salivary glands of rabies-virus-infected mice
Source: Front Microbiol. 2024 Feb 6;15:1354936. doi: 10.3389/fmicb.2024.1354936 (PMC10877373; doi:10.3389/fmicb.2024.1354936)
Supplement: Supplementary file 1 [file Table_1.DOCX]

Supplementary Material

Transcriptome Analysis of Rabies virus-infected Mice Salivary Glands

Xin Guo^1†^, Maolin Zhang^1†^, Ye Feng^2,3†^, Xiaomin Liu^1^, Chongyang Wang^1^, Yannan Zhang ^1^, Zichen Wang ^1^, Danwei Zhang ^1^ and Yidi Guo ^1*^

*** Correspondence:** Yidi Guo; guoyd@jlu.edu.cn

## Supplementary Figures:


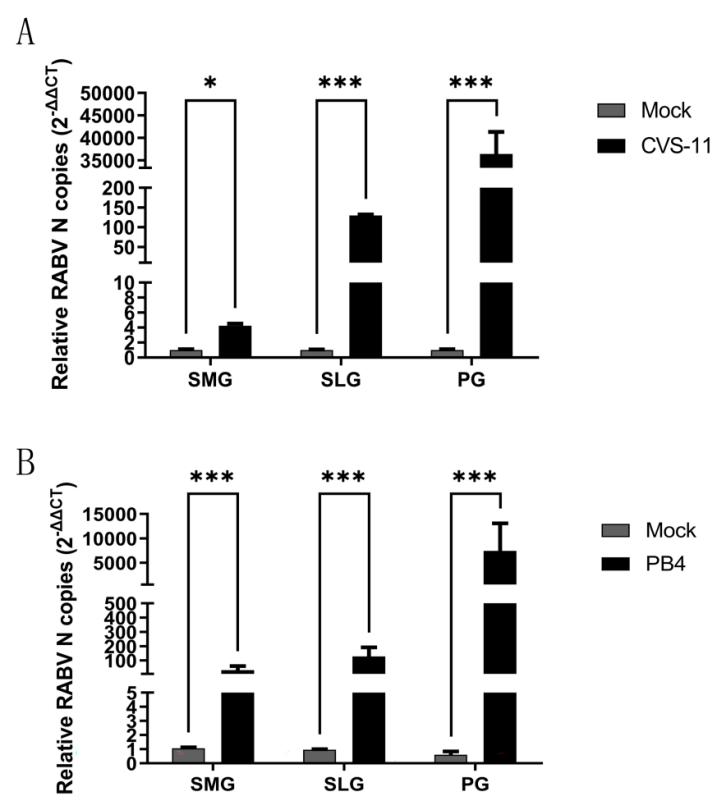


**SI Figure 1.** Effect of CVS-11 and PB4 infection on the three mice salivary glands: the submandibular gland (SMG), sublingual gland (SLG), and parotid gland (PG). The tissues infected with CVS-11 **(A)** and PB4 **(B)** were lysed to determine RABV N RNA copy numbers by RT-qPCR. Expression of all genes were normalized to house keeping gene of GAPDH. Student’s t test, p < 0.05 (*); p < 0.01 (**); p < 0.001 (***).
